# Robot-Assisted Low Anterior Resection Using the hinotori™ Surgical Robot System in a Patient with Situs Inversus Totalis: A Case Report

**DOI:** 10.70352/scrj.cr.26-0490

**Published:** 2026-11-10

**Authors:** Ryuichi Asai, Jesse Yu Tajima, Kento Terasaki, Ryoma Yokoi, Keita Matsumoto, Masahiro Fukada, Yuta Sato, Itaru Yasufuku, Yoshihiro Tanaka, Nobuhisa Matsuhashi

**Affiliations:** Department of Gastroenterological Surgery and Pediatric Surgery, Gifu University Graduate School of Medicine, Gifu, Gifu, Japan

**Keywords:** situs inversus totalis, hinotori, robot-assisted surgery, rectal cancer

## Abstract

**INTRODUCTION:**

Situs inversus totalis (SIT) is a rare congenital anomaly characterized by a complete mirror-image reversal of organs. Especially in a minimally invasive surgery, care must be taken to avoid misidentification because of reversed anatomical landmarks. Robot-assisted surgery (RAS) may be a feasible surgical option and may facilitate precise surgical manipulation in anatomically challenging situations. Although there are several reports of RAS using the da Vinci surgical robot system (Intuitive Surgical, Sunnyvale, CA, USA) for colorectal cancer (CRC) in patients with SIT, the optimal robot platform, port settings, and procedures have not been determined.

**CASE PRESENTATION:**

In a 74-year-old woman with SIT referred to our department for treatment of advanced rectal cancer, preoperative examinations showed cancer invasion beyond the intestinal walls and multiple lymph node swelling within the mesentery. After completing 4 cycles of preoperative chemotherapy, robot-assisted low anterior resection using the hinotori™ surgical robot system (hinotori; Medicaroid, Kobe, Hyogo, Japan) was planned. Prior to the surgery, we conducted meticulous simulation with other medical staff using the hinotori, instruments, monitors, and a dry box. During the operation, port placements and instruments were set in a completely left–right reversed position from the standard configuration. From the medial approach through mesenteric vessel division and dissection of the left-sided colon and rectosigmoid junction, the cockpit surgeon smoothly operated monopolar curved scissors with the non-dominant left hand with reversed procedures. In the operating room, a monitor displaying a left–right flipped image was placed alongside the original-image monitor, and surgery proceeded while the patient-side surgeons confirmed anatomical accuracy. As the hinotori has no sealing devices or linear staplers, vessel ligation, division of the mesorectum, and rectal transection were performed by the patient-side surgeons. The patient was discharged without major complications.

**CONCLUSIONS:**

To our knowledge, this is the first report of a surgical robot system other than the da Vinci system used to perform surgery for CRC in a patient with SIT. Sufficient preoperative simulation and display of flipped images contributed to the safe operation of robot-assisted low anterior resection in the patient. It is necessary to understand the characteristics of each surgical robot platform and perform surgery by leveraging the system’s individual features.

## Abbreviations

CAPOX
: capecitabine and oxaliplatin
CRC
: colorectal cancer
LS
: laparoscopic surgery
MCS
: monopolar curved scissors
MIS
: minimally invasive surgery
RAS
: robot-assisted surgery
SIT
: situs inversus totalis

## INTRODUCTION

SIT, a rare congenital malformation estimated to occur in approximately one in 5000 to 10000 people, is characterized by left–right reversal of organ positions.^1)^ Although functional issues are often absent, one must exercise caution to avoid anatomical misidentification, particularly in MIS. There are several reports of LS and RAS for CRC in patients with SIT; however, they remain limited.^2–14)^ Furthermore, all previous reports of RAS for CRC in patients with SIT have exclusively used the da Vinci surgical robot system (da Vinci; Intuitive Surgical, Sunnyvale, CA, USA).^9–13)^ The hinotori™ surgical robot system (hinotori; Medicaroid Corporation, Kobe, Hyogo, Japan) is the first surgical robot system domestically developed in Japan. It features 8-axis arms that closely resemble human arms and utilizes a free-docking system. Its slender arms provide spacious workspace for the patient-side surgeon. In this report, we describe the clinical course and surgical procedures of robot-assisted low anterior resection using the hinotori in a patient with SIT.

## CASE PRESENTATION

A 74-year-old woman was diagnosed as having SIT since childhood. Following a positive fecal occult blood test, rectal cancer was detected through colonoscopy, and she was referred to our hospital for treatment. The patient suffered from hypertension and was obese, with a height of 152.4 cm, weight of 73.0 kg, and BMI of 31.4 kg/m^2^. Chest X-ray showed dextrocardia, suggesting situs inversus (**Fig. 1A**). Colonoscopy revealed a tumor in the upper rectum, and biopsy confirmed diagnosis of moderately differentiated tubular adenocarcinoma (**Fig. 1B**). Contrast-enhanced CT with 3D angiography showed wall thickening with contrast enhancement in the upper rectum, and complete left–right transposition of the abdominal viscera and vessels (**Fig. 1C**). No abnormalities in the branching or course of the abdominal vessels and ureters were observed, except for left–right transposition (**Fig. 1D**). Blood tests showed carcinoembryonic antigen and carbohydrate antigen 19–9 levels within the normal range. Based on the above findings, the patient was diagnosed as having T3N2aM0 Stage IIIB (UICC 8th edition) cancer. Four cycles of CAPOX chemotherapy were administered preoperatively. Although the tumor shrank, residual tumor was detected, and robot-assisted curative resection using the hinotori was planned. Given that the patient had SIT, we determined that careful consideration of the robot’s roll-in direction, port placements, and instruments to be used, as well as information sharing across disciplines, would be necessary. Therefore, we conducted simulations using the hinotori, instruments, monitors, and a dry box in collaboration with surgical nurses and clinical engineers to ensure thorough preparation before proceeding with the surgery (**Fig. 2**).

**Fig. 1:**
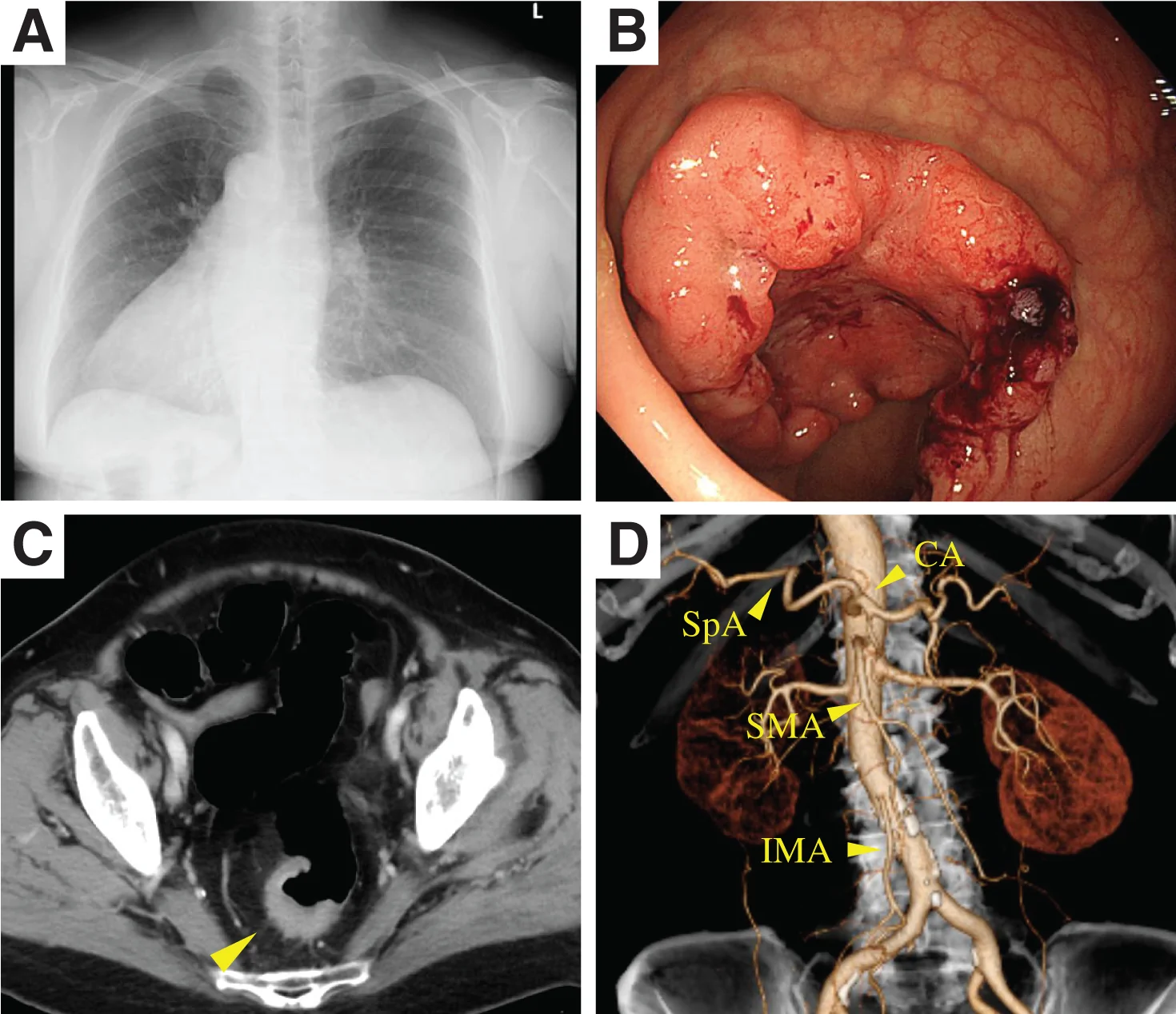
Preoperative examinations. (**A**) Chest X-ray showed dextrocardia, suggesting situs inversus. (**B**) Colonoscopy revealed an advanced rectal cancer in the upper rectum. (**C**) Enhanced CT scan revealed rectal wall thickening due to cancer in the pelvis (yellow arrowhead). (**D**) 3D CT angiography demonstrated complete left–right transposition of the abdominal viscera and vessels. CA, celiac artery; IMA inferior mesenteric artery; SMA, superior mesenteric artery; SpA, splenic artery

**Fig. 2:**
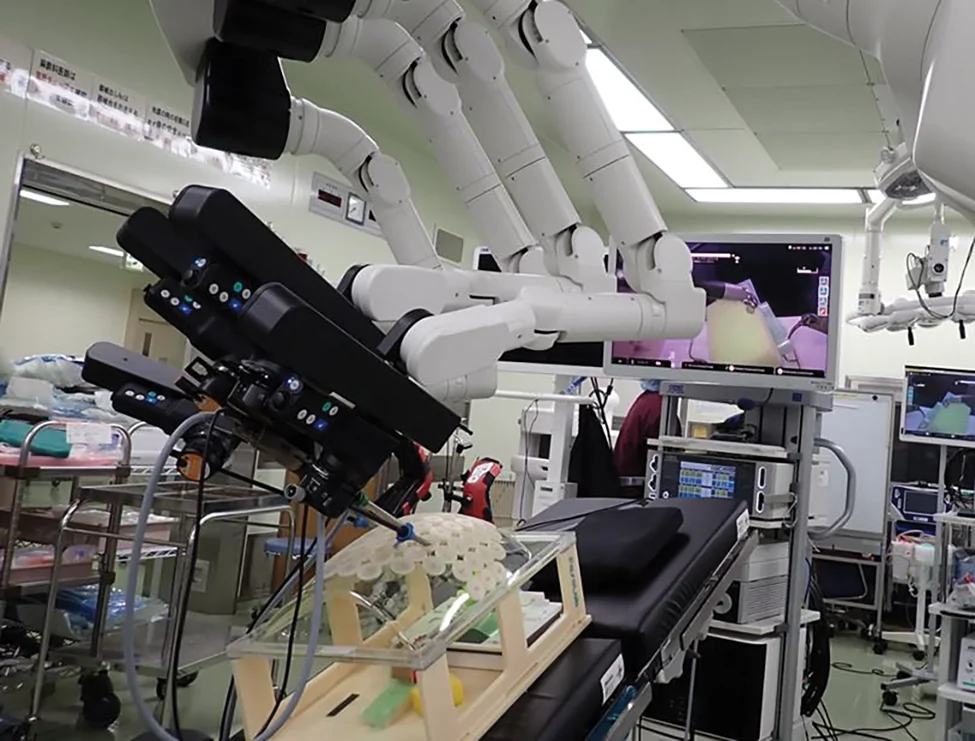
Preoperative photograph of simulation with medical staff using the hinotori™ (Medicaroid, Kobe, Hyogo, Japan), instruments, monitors, and a dry box.

During the surgery, the patient was placed in the Trendelenburg position at 17-degree and 7-degree tilted left-side-down. Port placements were performed by completely reversing the port configuration used for robot-assisted rectal resection in our department (**Fig. 3A** and **3B**). A 7 cm Lap Protector and E-Z ACCESS cap (both, Hakko, Chikuma, Nagano, Japan) were attached to the umbilicus, and a 10 mm robotic metal port for a 30-degree camera and 12 mm assist port were placed. Three robot ports were placed from the left lower abdomen to the right upper abdomen in a straight line. For the patient-side surgeons, 12 and 5 mm assist ports were placed at the left upper abdomen and right lower abdomen, respectively. After rolling in the hinotori from the patient’s right side, the arm base of the hinotori was tilted 7-degree left-side-down. We performed pivoting of Arm 3 for the camera, adjusting the arm positions automatically, and pivoting of the other arms to set the fulcrum. During the abdominal phase, which included the medial approach, mesenteric vessel division, left-sided colon mobilization, and dissection around the rectosigmoid junction, we attached croce grasping forceps to Arm 1, MCS to Arm 2, the camera to Arm 3, and bipolar fenestrated forceps to Arm 4. These instruments were also placed in completely left–right reversed positions from our standard configuration. During the pelvic phase, which included rectal dissection, division of the mesorectum, we intentionally changed the instrument configuration by placing the bipolar fenestrated forceps on Arm 2 and the MCS on Arm 4 (**Fig. 3C**). In the operating room, a monitor displaying the left–right flipped image was placed alongside the original-image monitor (**Fig. 4**). During the abdominal phase, the cockpit surgeon carefully operated the MCS with his non-dominant left hand while performing surgical maneuvers in a fully reversed configuration including forceps manipulation and tissue retraction (**Fig. 5A** and **5B**). This procedure minimized interference between the instruments and operational constraints. The surgery was performed while the patient-side surgeons confirmed that the anatomical orientation matched that of a standard procedure by checking a flipped monitor. For the pelvic phase, we attached bipolar fenestrated forceps to Arm 2 and the MCS to Arm 4 and performed the surgery with the cockpit surgeon using his dominant right hand as the primary manipulator because the anatomy is bilaterally symmetrical in the pelvis (**Fig. 5C**). As the hinotori does not have sealing devices or linear staplers, the patient-side surgeons inserted these devices through the assist port to divide the mesorectum and perform rectal transection (**Fig. 5D** and **5E**). After roll-out of the hinotori, the specimen was extracted through the umbilical incision. Finally, end-to-end anastomosis was laparoscopically completed with double stapling technique (**Fig. 5F**). A **Supplementary Video 1** is provided to demonstrate the surgical procedure, including the left–right flipped image and the corresponding modifications in instrument configuration and surgical maneuvers. The total operative time was 332 minutes, with 206 minutes spent at the console. Blood loss was minimal. Although a Clavien–Dindo grade I surgical site infection occurred at the umbilicus, the patient was discharged on the 11th POD. Postoperative pathological diagnosis revealed T3N1aM0 Stage IIIB cancer. The patient underwent 4 additional cycles of CAPOX chemotherapy and remains recurrence-free at 6 months postoperatively.

**Fig. 3:**
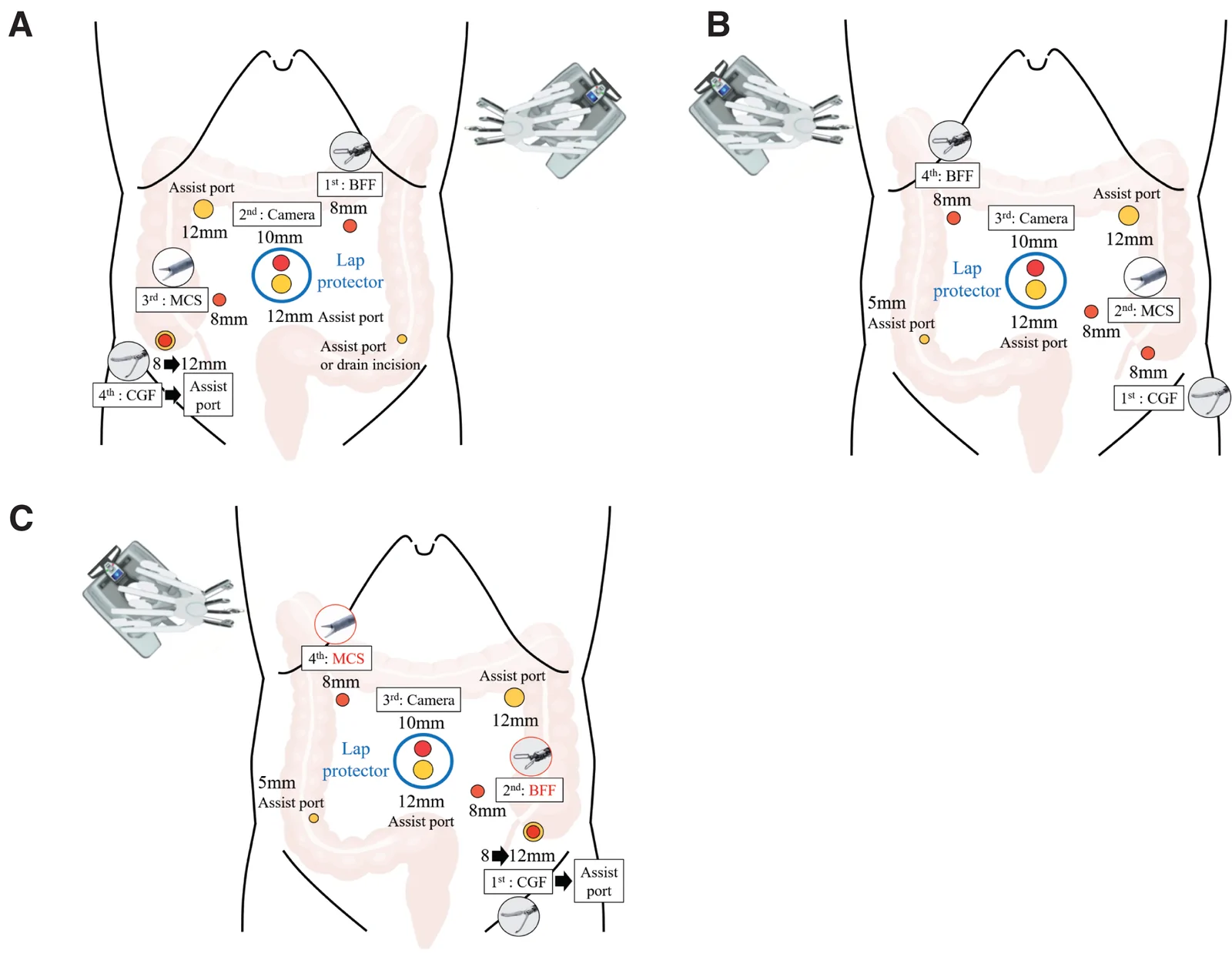
Configuration of the ports and instruments. (**A**) Standard configuration in our department without SIT. (**B**) Configuration during the abdominal phase (medial approach, mesenteric vessel division, left-sided colon mobilization, and dissection around the rectosigmoid junction). (**C**) Configuration during the pelvic phase (rectal dissection and division of the mesorectum). Red circle, robot port. Yellow circle, assist port. BFF, bipolar fenestrated forceps; CGF, croce grasping forceps; MCS, monopolar curved scissors; SIT, situs inversus totalis

**Fig. 4:**
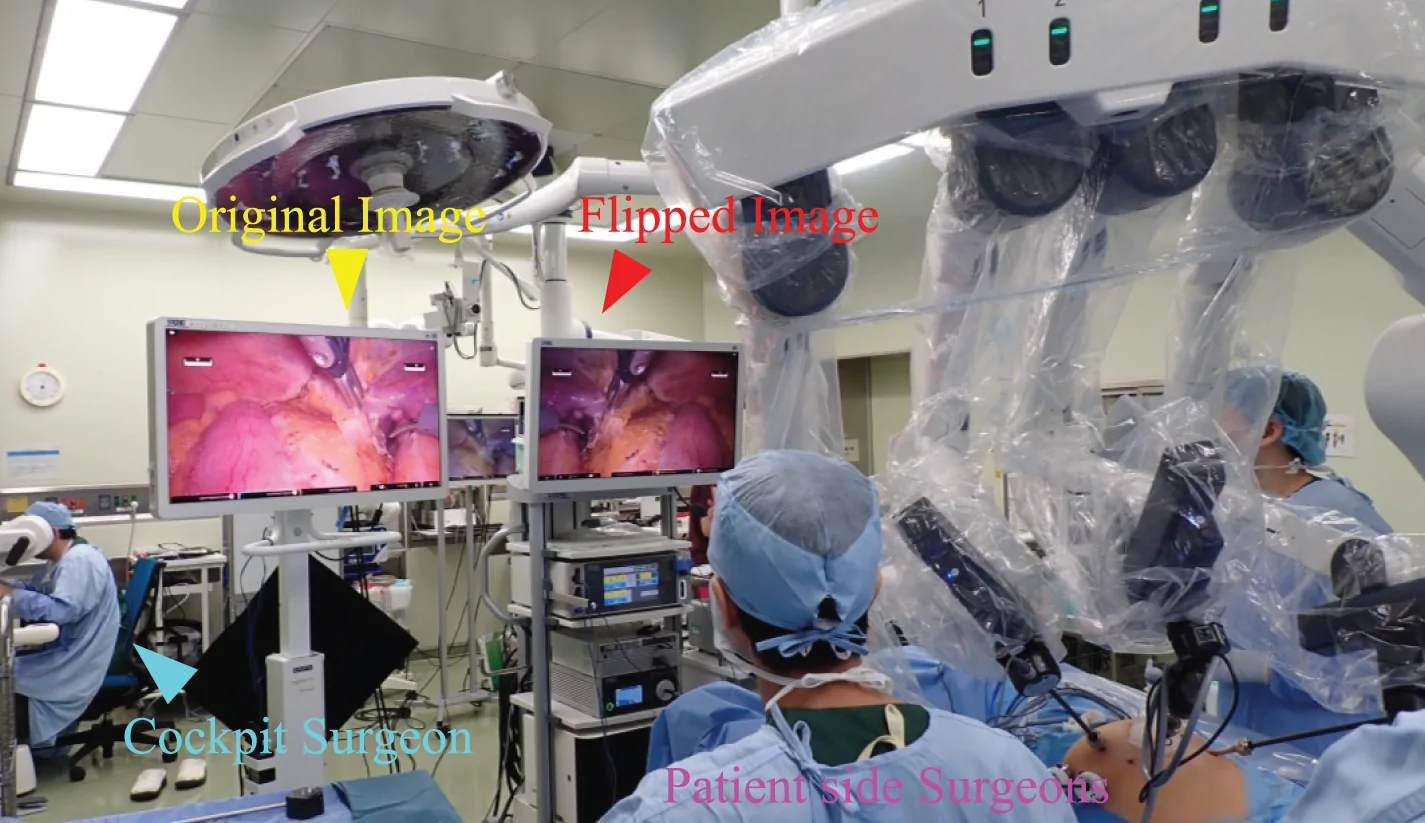
Operation room with surgeons and monitors.

**Fig. 5:**
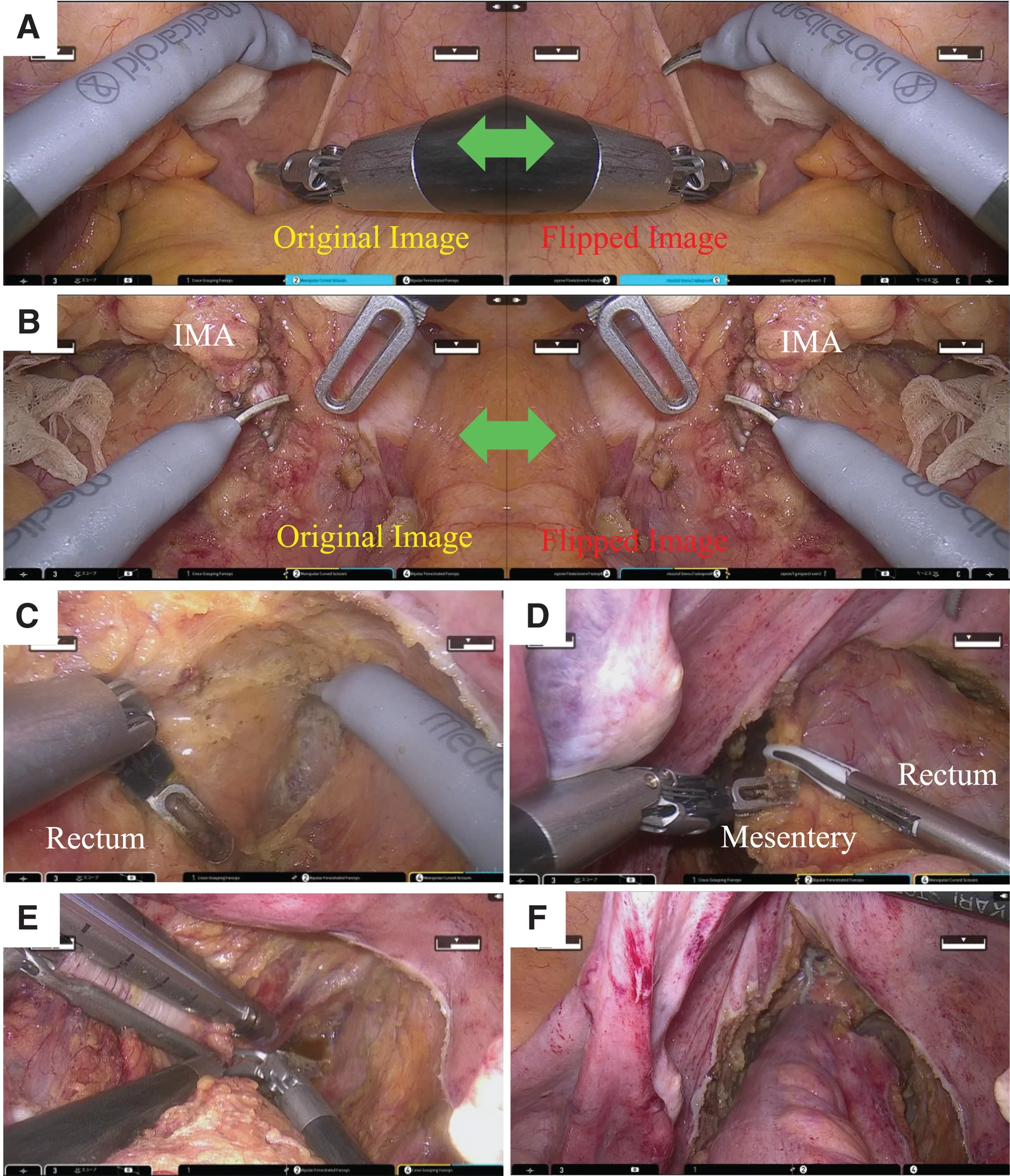
Intraoperative photographs. Original and flipped images of (**A**) peritoneal incision on the dorsal side of the IMA and (**B**) transection of the IMA after ligation of its root. Original images of (**C**) dissection of the right side of the rectum, (**D**) division of the mesorectum by the patient-side surgeon, (**E**) rectal transection by linear stapler, and (**F**) completion of the anastomosis. IMA, inferior mesenteric artery

## DISCUSSION

In recent years, reports of MIS for CRC in patients with SIT have begun to emerge, along with an increase in reports on RAS. Previous reports on MIS for rectal cancer in SIT are summarized in **Table 1**.^2–13)^ Although RAS tends to require more ports, previous studies have reported comparable operative times and short-term outcomes between RAS and LS, suggesting that RAS may be a feasible surgical option. Port and instrument placement are key considerations in RAS. Reported port placements in RAS for rectal or anal fistula cancer include the square position,^9–11)^ left–right straight position,^12)^ and left lower-right upper abdomen straight position, which is a complete mirror position like the one used in the present case,^13,15)^ indicating that no single standard was established. Minami et al. have summarized the advantages and disadvantages of LS and RAS in patients with SIT based on previous reports.^14)^ They report that in LS, it is often impossible to perform surgical maneuvers in the same manner as in standard anatomy, and dissection frequently requires the use of the non-dominant hand. Consequently, the difficulty level increases and operative time is longer compared to standard cases without SIT. However, they noted that RAS allows for precise dissection and can be safely performed due to the robot’s multi-jointed function with stable instruments and camera control. While 1 study reports that left-handed surgeons may find LS easier to perform in patients with SIT,^16)^ other reports indicate that RAS offers a wide range of motion and high freedom of movement regardless of the surgeon’s dominant hand.^12,16)^ However, there are natural limitations to the range of motion in RAS because fixed ports are used. Docking and targeting may need to be performed twice, or instruments may have to be changed unexpectedly during the procedure. Kita et al. reported this problem during an abdominoperineal resection for anal fistula cancer in a patient with SIT.^15)^ They initially started the procedure using the MCS attached to Arm 4 in the right upper abdomen and operated it with the right hand. However, when they reached the area near the root of the inferior mesenteric artery, the instruments became restricted. Then, they switched the MCS to Arm 2 in the left lower abdomen, which allowed for smooth dissection. In the present case, throughout the abdominal phase, we configured not only the ports in the reversed positions but also the instruments and procedures for the reversed positions and steps. We performed the surgery using the MCS controlled by the surgeon’s non-dominant left hand, and assigned each instrument a role nearly identical to that in the same manner without SIT. Operating the MCS with the non-dominant hand resulted in reduced precision and stability, thereby slowing instrument manipulation. However, with careful manipulation, the procedure can still be performed smoothly without substantial additional time, owing to the inherent stability and tremor filtration of RAS. During the pelvic phase, where the anatomy is nearly symmetrical, we intentionally switched the instrument configuration to operate the MCS with the dominant hand, enabling more efficient instrument manipulation. In the present case, no unintended instruments changes were necessary. We believe this technique was accomplished because of the surgical robot’s multi-jointed function and its precise, tremor-free, and stable operation. Therefore, left–right reversed configurations and procedures may be an option for RAS in patients with SIT.

**Table 1:** Previous reports of MIS for rectal cancer in patients with SIT

	Type of MIS (robot systems)	Year	Author	Age/Sex	BMI (kg/m^2^)	Operative procedure	Port placement	Number of incisions	Docking	Operative time (minutes)	Blood loss (mL)	pTNM	Posmiddleerative hospital stay (days)	Complications
1	Laparoscopy	2010	Huh et al.^2)^	41/female	23.0	LAR	Square	5	—	250	120	T3N0M0	8	None
2	Laparoscopy	2015	Fang et al.^3)^	39/female	19.7	LAR	Square	4	—	175	10	T2N0M0	7	None
3	Laparoscopy	2015	Ito et al.^4)^	53/female	26.5	APR	Square	5	—	380	390	T3N1aM0	27	None
4	Laparoscopy	2015	Choi et al.^5)^	43/male	—	APR	Square	4	—	325	300	T3N0M0	10	None
5	Laparoscopy	2020	Chen et al.^6)^	68/female	—	LAR with ileostomy	—	—	—	—	—	T3N0M0	—	None
6	Laparoscopy	2024	Song et al.^7)^	61/male	—	Hartmann’s procedure	Square	5	—	—	—	T3N0M0	—	None
7	Laparoscopy	2025	Miyasaka et al.^8)^	81/male	—	LAR using transanal approach	Single	1	—	228	—	T3N0M0	15	None
8	Robot (da Vinci^a^)	2012	Leong et al.^9)^	70/female	—	LAR with ileostomy	Square*	6	Single	—	—	T3N0M0	6	None
9	Robot (da Vinci S^b^)	2015	Foo and Law^10)^	59/male	—	LAR	Square*	6	Dual	204	100	T3N2M1	4	None
10	Robot (da Vinci)	2018	Cui et al.^11)^	61/male	—	LAR with transanal NOSE	Square*	5	Single	210	50	T2N0M0	7	None
11	Robot (da Vinci Xi^c^)	2021	Kasai et al.^12)^	60s/female	21.4	HAR	Straight* (Left–right)	6	Dual	194	Minimal	T3N2bM0	6	None
12	Robot (da Vinci Xi)	2026	Takaoka et al.^13)^	71/male	—	LAR	Straight* (LLA–RUA)	5	Single	304	5	T3N1M0	—	None
13	Robot (hinotori™^d^)	2026	Our case	74/female	31.4	LAR	Straight* (LLA–RUA)	6	Single	332	Minimal	T3N1aM0	11	SSI: Grade I^†^

*Placement of robotic ports. ^†^Clavien-Dindo classification. ^a^Intuitive Surgical, Sunnyvale, CA, USA. ^b^Intuitive Surgical. ^c^Intuitive Surgical. ^d^Medicaroid, Kobe, Hyogo Japan.

APR, abdominoperitoneal resection; HAR, high anterior resection; LAR, low anterior resection; LLA, left lower abdomen; MIS, minimally invasive surgery; NOSE, natural orifice specimen extraction; pTNM, pathologic tumor-node-metastasis; RUA, right upper abdomen; SIT, situs inversus totalis; SSI surgical site infection

Surgery in patients with SIT carries a risk of port placement errors due to anatomical inversion and intraoperative misidentification of anatomical structures. Therefore, prior simulation and preparatory measures are crucial to ensure safe surgical procedures. Consistent with another report, we also conducted simulations using an actual robotic system in collaboration with surgical nurses and clinical engineers in a dry box. Before proceeding with the actual surgery, we thoroughly evaluated port placement, the instruments to be used, and their positions.^15)^ Furthermore, we previously reported the usefulness of a left–right flipped monitor for appendiceal tumor in SIT.^17)^ In the present case as well, by preparing a flipped image during the simulation phase and using it during the procedures, we were able to perform the surgery safely. Because the instruments and procedures were also totally reversed throughout the abdominal phase, the flipped images showed that the positions of the instruments and anatomical landmarks matched those of a standard procedure without SIT. This made it easy to confirm that the correct maneuvers were being performed. However, it should be noted that patient-side surgeons may become confused when viewing the flipped images. Measures such as appropriate monitor positioning or clear labeling may help prevent confusion between the original and flipped images. In RAS, close communication and careful coordination between the cockpit surgeon and patient-side surgeons are important, particularly in atypical cases such as those involving SIT.

The hinotori is the first surgical robot system domestically developed in Japan. Miura et al. first reported its use for rectal cancer in 2023,^18)^ and it is reported to result in fewer subcutaneous emphysemas compared to the da Vinci Xi during surgery for CRC.^19)^ The use of a free-docking system in which the robotic arms are not fixed to ports allows for slimmer robotic arms and wider spacing between them, thus providing patient-side surgeons with ample room for maneuvers. The present case represents, to our knowledge, the first report of RAS performed on a patient with SIT using a surgical robot system other than the da Vinci. Although clinical evidence for the hinotori remains limited compared with that for the da Vinci, both platforms share several fundamental features, suggesting that the hinotori may be applicable to similar surgical procedures. On the other hand, in our experience, the hinotori may be more susceptible to motion limitations than the da Vinci when operating near the limits of its working range. As mentioned above, even with the da Vinci Xi, Kita et al. reported an unplanned instrument exchange near the root of the inferior mesenteric artery due to limited instrument mobility when the MCS was positioned in the right upper abdomen.^15)^ Therefore, the hinotori may be well suited for surgical procedures performed in a reverse configuration, as in the present case. Since the hinotori does not have sealing devices or linear staplers, the patient-side surgeons performed vessel ligation, mesorectal division, and manipulation of the linear stapler. One of the distinctive features of the hinotori is the ample working space available for the assistant, which allowed these procedures to be performed smoothly without interference from the robotic arms. Although this report does not establish the superiority of the hinotori, it demonstrates the feasibility of surgery in patients with SIT using surgical robotic systems other than the da Vinci by leveraging the specific characteristics of each system.

## CONCLUSIONS

We performed low anterior resection using the hinotori for a patient with SIT. In addition to the port configuration, the instrument settings and surgical maneuvers were completely reversed from the beginning of the robotic procedure through dissection around the rectosigmoid junction. The surgery was completed safely with the patient experiencing a favorable clinical course. Although RAS may be safely performed in patients with SIT, adequate preparation and intraoperative safety precautions, such as preoperative simulation and display of flipped images during surgery, are essential. Furthermore, it is essential to understand the characteristics of each surgical robot system and perform surgery by leveraging the system’s individual features.

## SUPPLEMENTARY MATERIALS

Video 1 Robot-assisted low anterior resection using the hinotori surgical robot system in a patient with situs inversus totalis: a surgical video with left–right flipped images.
